# Biomarker candidates for cardiovascular disease and bone metabolism disorders in chronic kidney disease: a systems biology perspective

**DOI:** 10.1111/j.1582-4934.2008.00280.x

**Published:** 2008-02-08

**Authors:** Paul Perco, Julia Wilflingseder, Andreas Bernthaler, Martin Wiesinger, Michael Rudnicki, Barbara Wimmer, Bernd Mayer, Rainer Oberbauer

**Affiliations:** a Medical University of Vienna, Währinger GürtelVienna, Austria; b Krankenhaus der Elisabethinen, FadingerstrasseLinz, Austria; c Institute for Theoretical Chemistry, University of ViennaWähringer Strasse, Vienna, Austria; d Medical University of InnsbruckAnichstrasse, Innsbruck, Austria; e Emergentec Biodevelopment GmbHRathausstrasse, Vienna, Austria

**Keywords:** proteomics, genomics, systems biology, chronic kidney disease, cardiovascular disease, bone metabolism disorders

## Abstract

Patients with chronic kidney disease (CKD) show a panel of partially de-regulated serum markers indicative for bone metabolism disorders and cardiovascular diseases (CVDs). This review provides an overview of currently reported biomarker candidates at the interface of kidney disease, bone metabolism disorders and CVDs, and gives details on their functional interplay on the level of protein–protein interaction data. We retrieved 13 publications from 1999 to 2006 reporting 31 genes associated with CVDs, and 46 genes associated with bone metabolism disorders in patients with CKD. We identified these genes to be functionally involved in signal transduction processes, cell communication, immunity and defence, as well as skeletal development. On the basis of the given set of 77 genes further 276 interacting proteins were identified using reference data on known protein interactions. Their functional interplay was estimated by linking properties reflected by gene expression data characterizing CKD, gene ontology terms as provided by the gene ontology consortium and transcription factor binding site profiles. Highly connected sub-networks of proteins associated with CKD, CVDs or bone metabolism disorders were detected involving proteins like collagens (COL1A1, COL1A2), fibronectin, transforming growth factor-β_1_, or components of fibrinogen (FG-α, FG-β, FG-γ). A systems biology approach provides a methodological framework for linking singular biomarker candidates towards deriving functional dependencies among clinically interlinked diseases.

- Introduction
- Kidney disease and cardiovascular risk
- Kidney disease and bone metabolism disorders
- Data integration and systems biology analyses
  - Data preparation
  - Functional annotation
  - Protein–protein interaction network analysis
  - Integrated analysis
- Conclusion and outlook

## Introduction

The use of serum biomarkers has been successfully demonstrated in the clinical context of cardiovascular diseases (CVDs) and bone metabolism disorders, and their predictive value as well as discriminatory power has been well established [1]. Both – CVDs and bone metabolism disorders – might be causally linked in patients with chronic kidney disease (CKD), because the disturbances of the calcium-, phosphate-, vitamin D- and parathyroid hormone (PTH) metabolism, as well as the incidence of cardiovascular events as myocardial infarction rises early in the course of kidney disease [2, 3].

Next to established diagnostic and prognostic parameters new biomarker candidates are currently arising with astonishing speed, in particular facilitated by genomic and proteomic techniques allowing scans of whole transcriptomes and proteomes of clinical samples. Experimental procedures for deriving such initial marker profiles have traversed towards a routine procedure. The tough part, however, is the choice of those candidates with clinical relevance for further validation studies [4, 5]. Data integration, bioinformatics analyses and functional testing of novel hypotheses drawn have been identified as a valuable strategy, commonly denoted in the context of systems biology [6].

Mondry and colleagues emphasized the potential of systems biology and quantitative models in their review on the molecular mechanisms of renal osteodystrophy [7]. Drake *et al.* focused on proteomic approaches and the use of protein–protein interaction data for biomarker discovery in their review on systems biology of CVDs [8].

This review provides an overview on the suspected link between CVDs and bone metabolism disorders in patients with impaired renal function, and will furthermore characterize and analyse reported biomarkers associated with these particular diseases. Subsequently, the interdependency of reported biomarkers will be analysed on a systems biology level taking into account data on gene expression in CKD, functional gene annotation, protein–protein interactions, as well as gene regulatory elements reflected by joint transcription factor binding sites.

## Kidney disease and cardiovascular risk

CKD is associated with increased risk for cardiovascular complications and all cause mortality. The risk of death and the prevalence of CVD start to rise significantly already in patients with early stage renal insufficiency, *i.e.* with a glomerular filtration rate (GFR) of less than 60 ml/min. [9]. In dialysis patients the prevalence of CVD and the mortality due to CVD is even 10 to 30 times higher than in the general population [10]. Cardiovascular events in CKD patients are caused by traditional and non-traditional risk factors and their interactions: Atherosclerosis, arteriosclerosis and altered cardiac morphological characteristics are the main findings [11]. These complex characteristics impose a new challenge in identifying and treating patients with CVD in early stages of CKD towards improving outcome. So far there are no validated biomarkers for identifying the risk of CVD in CKD patients available. As for all bio-markers, CVD markers should be easily measurable and significantly de-regulated in disease states. In statistical terms this constraint refers to adequate discrimination (receiver operating characteristic–area under curve [ROC–AUC]), as well as transportability, *i.e.* validity of a marker in different patient populations. The cardiovascular biomarkers which are discussed in the paper by Roberts *et al.* are involved in several pathophysiological processes such as endothelial dysfunction, vascular calcification, monocyte recruitment to the endothelium, inflammation, oxidative stress, sympathetic nervous system activation, glycosylation of proteins, bone marrow function, platelet activation, left ventricular structure and function, myocardial necrosis and other processes [11]. According to the authors an improvement in cardiovascular risk stratification might be achieved by measuring a combination of cardiovascular biomarkers, each representing a different aspect of CVD pathophysiology. Next to their function for assessing the level of risk of vascular disease, biomarkers could depict potential targets for the prevention of such disease [12]. However, the link between the given CVD biomarker candidates and CKD remains elusive.

## Kidney disease and bone metabolism disorders

The kidney is involved in calcium/phosphate homeostasis which is tightly regulated by the phosphate-excretion regulating hormones (phosphatonins) fibroblast growth factor 23 (FGF23), PTH and by the action of the active form of vitamin D (1α,25-dihydroxy cholecal-ciferol or calcitriol) which is exclusively synthesized in the kidney. In the healthy individual PTH is secreted by the parathyroidea upon hypocalcemia and/or hyperphosphatemia. PTH stimulates the release of calcium and phosphate from bone tissue, the synthesis of calcitriol in the kidney and the reabsorption of calcium by the enteric mucosa and the distale tubule cells in the kidney. On the other hand, PTH increases the excretion of phosphate in by the renal proximal tubule cells. The renal 1-α hydroxylation of vitamin D which transforms vitamin D into its active form calcitriol is PTH dependent. Calcitriol increases the enteral and renal calcium and phosphate reabsorption and bone mineralization. Finally, FGF23 is a phosphatonin increased by high phosphate ingestion. FGF23 enhances fractional renal phosphate excretion and inhibits the 1-α hydroxylation of vitamin D, thus directly interfering with calcitriol synthesis.

In stages I and II of kidney disease, *i.e.* when GFR is normal or only slightly reduced, the levels of calcium, phosphate and PTH in plasma are usually not different from healthy individuals [13]. During progression to stage III of CKD, fractional renal excretion of phosphate rises mainly due to phosphate retention and subsequent increased levels of the phosphatonins FGF23 and PTH, which keep the serum phosphate levels in the normal range [14]. Thus progression of kidney disease causes changes in phosphate homeostasis finally leading to a rise in serum levels of PTH which is called secondary hyperparathyroidism (sHPT). In addition patients with stage III kidney disease frequently suffer from a deficiency in 25-OH-vitamin D_3_, which leads to diminished synthesis of active vitamin D_3_[15]. Furthermore, the action of the 1-α-hydroxy-lase in the kidney is inhibited by rising levels of FGF23 and by the progression of renal insufficiency *per se*, which finally leads to decreased plasma levels of active vitamin D3 [14]. If GFR falls below 30 ml/min. (*i.e.* stage IV and V of chronic kidney disease) the excretion of phosphate cannot be enhanced any further and hyperphos-phatemia develops.

sHPT leads to severe changes in bone mineralization and structure, and the term chronic kidney disease–mineral bone disorder (CKD–MBD) was coined [16, 17]. However, PTH receptors are not only found in kidney, bone and enteric mucosal cells but also in the cardiovascular system. Therefore, sHPT and adjacent vitamin D therapy not only lead to CKD–MBD but is also associated with the development of vascular, valvular and extravascular calcifications, all increasing mortality [18].

The degree of bone formation rate can be somehow estimated by determining plasma levels of several marker proteins. Although the plasma levels of bone-specific alkaline phosphatase, osteocalcin and procollagen type I carboxy-terminal extension peptide stand for the degree of bone formation, the bone resorption rate is represented, *e.g.* by procollagen type I crosslinked carboxy-terminal telopeptide, plasma deoxypyridinoline, bone-specific tartrateresistant acid phosphatase and some of the multiple products resulting from the degradation of type I collagen [19, 20]. Other circulating molecules are of growing interest as they may also be indicative for the bone turnover rate, namely osteoprotegrin, bone sialoprotein, β_2_-microglobulin, cathepsins, nitric oxide, advanced oxidation protein products, advanced glycation products, cytokines as interleukines (mostly IL-1, IL-6 and IL-11), soluble IL-6 receptor, tumour necrosis factor-α (TNF-α), transforming growth factor-β (TGF-β), bone morphogenetic proteins (BMPs) and their soluble receptors, growth factors such as insulin growth factor-1 (IGF-1), macrophage colony stimulating factor and gran-ulocyte-macrophage colony stimulating factor [17, 20].

About 30% of patients with end stage renal failure exhibit coronary heart disease (USRDS registry ‘Annual Data Report 2007’http://www.usrds.org/). At the same time, almost all patients with advanced renal impairment show a multifactorial bone disease [17, 21]. Progression of each of the three entities is strongest when the other two organ systems are malfunctioning. Furthermore, it has recently been shown by a worldwide multi-centre trial, that traditional markers and risk factors for CVD in the general population such as hypercholesterolaemia, arterial hypertension or elevated body mass index exhibit a U-shaped association with cardiac events in patients with end stage renal disease [22, 23].

Thus based on this evidence the review sought to elucidate the current knowledge of molecular markers to uncover and correctly classify the individual risk for this dangerous triad. By identifying patients at risk, potential prophylactic and/or therapeutic measure might be taken in time before end organ failure is clinically evident.

## Data integration and systems biology analyses

### Data preparation

Peer reviewed publications (PubMed, http://www.ncbi.nlm.nih.gov/, status as of December 2006) were screened for genes or proteins associated with CVDs or bone metabolism disorders in chronic kidney patients. The following keywords were used during the literature search: ‘biomarker(s)’, ‘risk factor(s)’, ‘chronic kidney disease’, ‘renal disease’, ‘cardiovascular disease’, ‘cardiovascular risk’ and ‘bone metabolism disorder’. Thirteen publications from 1999 to 2006 covered a non-redundant set of in total 73 genes associated with either CVD (n = 31) or bone metabolism disorders (n = 46) in patients with CKD, as summarized in Tables 1 and 2, respectively. Both serum as well as tissue markers were included without any restrictions in respect to the method of detection.

**Table 1:** CVD markers in CKD

Gene symbol	Gene name	Gene ID	References
ADIPOQ	Adiponectin, C1Q and collagen domain containing	9370	[11]
AHSG	α-2-HS-glycoprotein	197	[11]
CCL2	Chemokine (C-C motif) ligand	6347	[11]
CD40LG	CD40 ligand (TNF superfamily, member 5, hyper-IgM syndrome)	959	[11]
CRP	C-reactive protein, pentraxin-related	1401	[11]
CST3	Cystatin C (amyloid angiopathy and cerebral haemorrhage)	1471	[24]
EDN1	Endothelin 1	1906	[25]
FGA	Fibrinogen, α chain	2243	[11]
FGB	Fibrinogen, β chain	2244	[11]
FGG	Fibrinogen, γ chain	2266	[11]
ICAM1	Intercellular adhesion molecule 1 (CD54), human rhinovirus receptor	3383	[11]
IL6	Interleukin-6 (interferon, β-2)	3569	[26, 27]
IL8	Interleukin-8	3576	[11]
LEP	Leptin (obesity homologue, mouse)	3952	[28]
LPA	Lipoprotein, Lp(a)	4018	[29]
MTHFR	5,10-methylenetetrahydrofolate reductase (NADPH)	4524	[29]
NPPB	Natriuretic peptide precursor B	4879	[11]
NPY	Neuropeptide γ	4852	[30]
PAPPA	Pregnancy-associated plasma protein A, pappalysin 1	5069	[11]
PTH	Parathyroid hormone	5741	[30]
RLN1	Relaxin 1	6013	[11]
RLN2	Relaxin 2	6019	[11]
RLN3	Relaxin 3	117579	[11]
SAA1	Serum amyloid A1	6288	[11]
SAA2	Serum amyloid A2	6289	[11]
SELE	Selectin E (endothelial adhesion molecule 1)	6401	[11]
SELP	Selectin P (granule membrane protein 140 kD, antigen CD62)	6403	[11]
TNF	Tumour necrosis factor (TNF superfamily, member 2)	7124	[11]
TNNI3	Troponin I type 3 (cardiac)	7137	[11]
TNNT2	Troponin T type 2 (cardiac)	7139	[11]
VCAM1	Vascular cell adhesion molecule 1	7412	[11]

**Table 2:** Bone markers in CKD

Gene symbol	Gene name	Gene ID	References
ACP5	Acid phosphatase 5, tartrate resistant	54	[17]
ALPL	Alkaline phosphatase, liver/bone/kidney	249	[19]
B2M	β-2-microglobulin	567	[17]
BGLAP	Bone γ-carboxyglutamate (gla) protein (osteocalcin)	632	[17]
BMP1	Bone morphogenetic protein 1	649	[20]
BMP10	Bone morphogenetic protein 10	27302	[20]
BMP15	Bone morphogenetic protein 15	9210	[20]
BMP2	Bone morphogenetic protein 2	650	[20]
BMP3	Bone morphogenetic protein 3 (osteogenic)	651	[20]
BMP4	Bone morphogenetic protein 4	652	[20]
BMP5	Bone morphogenetic protein 5	653	[20]
BMP6	Bone morphogenetic protein 6	654	[20]
BMP7	Bone morphogenetic protein 7 (osteogenic protein 1)	655	[17]
BMP8A	Bone morphogenetic protein 8a	353500	[20]
BMP8B	Bone morphogenetic protein 8b (osteogenic protein 2)	656	[20]
BMPR1A	Bone morphogenetic protein receptor, type IA	657	[20]
BMPR1B	Bone morphogenetic protein receptor, type IB	658	[20]
BMPR2	Bone morphogenetic protein receptor, type II (serine/threonine kinase)	659	[20]
COL1A1	Collagen, type I, α-1	1277	[17]
COL1A2	Collagen, type I, α-2	1278	[17]
CSF1	Colony stimulating factor 1 (macrophage)	1435	[20]
CSF2	Colony stimulating factor 2 (granulocyte-macrophage)	1437	[20]
CTSL	Cathepsin L	1514	[17]
FGF23	Fibroblast growth factor 23	8074	[16]
FN1	Fibronectin-1	2335	[20]
GDF5	Growth differentiation factor 5	8200	[31]
GDF6	Growth differentiation factor 6	392255	[31]
GDF7	Growth differentiation factor 7	151449	[31]
IFNG	Interferon, γ	3458	[20]
IGF-1	Insulin-like growth factor 1 (somatomedin C)	3479	[17]
IL11	Interleukin-11	3589	[20]
IL1A	Interleukin-1, α	3552	[20]
IL1B	Interleukin-1, β	3553	[20]
IL6	Interleukin-6 (interferon, β-2)	3569	[20]
LEP	Leptin (obesity homologue, mouse)	3952	[28]
MEPE	Matrix, extracellular phosphoglycoprotein with ASARM motif (bone)	56955	[17]
PHEX	Phosphate regulating endopeptidase homologue, X-linked (hypophosphatemia, vitamin D resistant rickets)	5251	[17]
PLAU	Plasminogen activator, urokinase	5328	[20]
PTGES2	Prostaglandin E synthase 2	80142	[20]
PTH	Parathyroid hormone	5741	[16]
SERPINE1	Serpin peptidase inhibitor, clade E (nexin, plasminogen activator inhibitor type 1), member 1	5054	[20]
SPARC	Secreted protein, acidic, cysteine-rich (osteonectin)	6678	[20]
SPP1	Secreted phosphoprotein 1 (osteopontin, bone	6696	[20]
	sialoprotein I, early T-lymphocyte activation 1)		
TGFB1	Transforming growth factor-β_1_ (Camurati-Engelmann disease)	7040	[20]
TNF	Tumour necrosis factor (TNF superfamily, member 2)	7124	[20]
TNFRSF11B	Tumour necrosis factor receptor superfamily, member 11b (osteoprotegerin)	4982	[17]

Four genes are proposed as markers for cardiovascular as well as bone metabolism disorders, namely the PTH, the TNF, leptin (LEP), as well as IL-6.

### Functional annotation

Functional categories as well as molecular pathways holding a significant number of genes were identified using the Gene Expression Data Analysis Tool of the PANTHER (protein analysis through evolutionary relationships) Classification System [32, 33], and are listed in Tables 3 and 4. In PANTHER, proteins are assigned to families and subfamilies of shared function with two main categories, namely molecular function and biological processes. Biological processes and molecular functions of our candidate genes were compared with the PANTHER-internal reference dataset holding all 25,431 currently annotated human genes. A chi-squared test including Bonferroni correction to account for multiple testing was applied to compare the ratio of expected to observed frequency of genes assigned to certain ontology categories. This procedure identifies if certain ontologies are over- or under-represented on the basis of the given gene lists.

**Table 3:** Functional classification of CVD markers

Biological process	REFLIST (25431)	CVD markers (31)	*P*-value
Immunity and defence	1318	14	3.58E-09
Blood circulation and gas exchange	89	5	2.56E-06
Cell communication	1213	11	1.46E-05
Signal transduction	3406	14	5.14E-04
Blood clotting	92	4	7.23E-04
Ligand-mediated signalling	421	6	2.10E-03
Cytokine and chemokine mediated signalling pathway	252	5	2.59E-03
Cell proliferation and differentiation	1028	7	6.10E-03
Apoptosis	531	5	1.33E-02
Cell surface receptor mediated signal transduction	1638	9	1.51E-02
**Molecular function**			
Signalling molecule	795	18	3.29E-18
Peptide hormone	102	9	8.05E-13
Extracellular matrix	384	4	3.42E-02
Cytokine	97	3	3.71E-02
Cell adhesion molecule	395	4	3.80E-02
**Pathway**			
Plasminogen activating cascade	21	4	1.88E-06
Blood coagulation	55	4	8.61E-05

**Table 4:** Functional classification of bone markers

Biological process	REFLIST (25431)	Bone markers (46)	*P*-value
Skeletal development	123	15	1.63E-21
Mesoderm development	551	17	7.12E-15
Developmental processes	2152	20	6.98E-09
Cell communication	1213	16	2.61E-08
Cytokine and chemokine mediated signalling pathway	252	9	1.44E-07
Ligand-mediated signalling	421	9	1.17E-05
Cell surface receptor mediated signal transduction	1638	15	1.48E-05
Signal transduction	3406	20	1.76E-05
Other receptor mediated signalling pathway	210	5	7.86E-03
Immunity and defence	1318	9	1.60E-02
Macrophage-mediated immunity	140	4	1.81E-02
**Molecular function**			
Signalling molecule	795	26	6.55E-26
Growth factor	125	9	2.52E-10
Cytokine	97	8	1.65E-09
Other signalling molecule	259	7	6.91E-05
Interleukin	34	3	6.95E-03
Extracellular matrix	384	5	1.86E-02
**Pathway**			
TGF-β signalling pathway	154	16	3.58E-22

For both diseases, CVD and bone metabolism disorders, genes involved in the category ‘signal transduction’ were predominant. Twenty out of the 46 bone metabolism disorder biomarker candidates and 14 out of the 31 CVD marker candidates were assigned to this functional category. The most significantly enriched biological processes in CVD have been identified as immunity and defence (14 genes), blood circulation and gas exchange (5 genes), as well as cell communication (11 genes). Because several bone morphogenetic proteins are in the list of bone metabolism markers, the most significantly enriched biological processes in bone metabolism disorders are skeletal development (15 genes), mesoderm development (17 genes) and developmental processes (20 genes). The complete listing of all significant biological processes, molecular functions and biological pathways of the 77 biomarker candidates is given in Tables 3 and 4 for cardiovascular and bone metabolism disorders, respectively.

### Protein–protein interaction network analysis

**Fig. 1:**
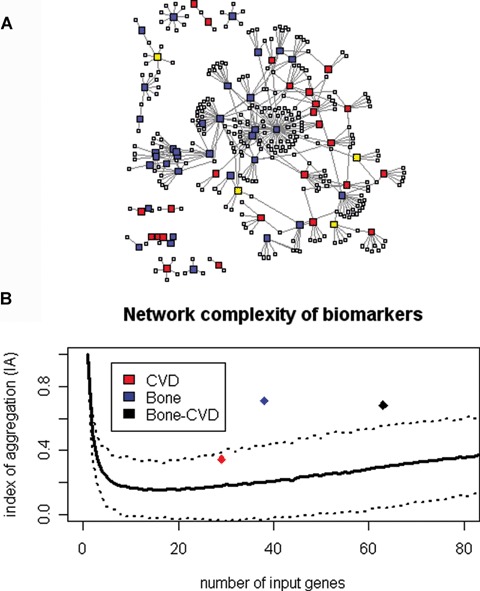
(**a**) Protein interaction networks after next neighbour expansion as given by the candidate biomarker lists of 77 proteins: Protein nodes given in blue depict bone markers, protein nodes given in red denote markers of cardiovascular disease, and protein nodes given in yellow represent proteins reported in both diseases. (**b**) Network complexity of protein interaction networks: Given is the index of aggregation (IA; y-axis) in relation to the number of proteins used for constructing protein interaction networks (x-axis). The IA of protein interaction networks derived on the basis of randomly generated protein lists is given as reference (solid line, dashed lines gives the standard deviation). The IA for networks based on the list of cardiovascular marker candidates alone does not exceed values also derived for randomly generated protein lists. The IA for networks derived for the given bone metabolism disorder markers, but in particular the combined markers significantly exceed reference values as found for randomly generated lists with the same number of proteins involved.

Next to identifying joint functional categories we used human protein–protein interaction data to determine the connectivity of the 77 biomarker candidates on the level of cellular protein networks. Human protein–protein interaction data from OPHID (Online Predicted Human Interaction Database) were used for the analysis (OPHID Version 2007-02-17) [34]. The generation of interaction networks followed the next neighbour expansion method as proposed by Chen *et al.*[35]. OPHID represents protein interactions as protein A interacts with protein B. If A and B are members of the list of 77 candidates a positive interaction is identified. The next neighbour expansion also includes interactions of the type A–X–B, where X represents a protein not given in the initial candidate list. All interacting partners of the initial set of 77 proteins were extracted from the OPHID database and the protein interaction network was generated. At least one interacting partner was found for 29 of the 31 CVD, and for 38 of the 46 bone metabolism markers. The resulting graph, composed of one large sub-graph and a number of smaller, disconnected sub-graphs, consisted of 353 protein nodes and 440 protein interaction edges, as depicted in Fig. 1a.

The index of aggregation (IA) serves as aggregation and complexity measure of interaction networks for evaluating if the interaction characteristics differ with respect to networks derived on the basis of random protein lists. This measure therefore gives an indication if the connectivity for a given protein list is higher than statistically expected. The IA is given as percentage of protein nodes in the largest sub-graph with respect to all protein nodes in the network including all sub-graphs. The IA of the biomarker candidates' network given in Fig. 1 was compared to respective values of randomly generated protein lists. Forty-three of the 63 proteins which actually have interaction entries in the OPHID database were connected in a single sub-graph when including next neighbour expansion. The resulting index of aggregation of 0.68 for the combined list of potential biomarkers (CVD and bone) is more than two standard deviations above the expected IA for randomly generated networks of equivalent size. Figure 1b shows the IA of the given biomarker lists in comparison to the distribution of the IA for randomly generated protein lists. Genes associated with CVDs and those associated with bone metabolism disorders are highly interlinked on the level of protein–protein interactions.

Both, functional categories as well as protein interactions indicate the interrelation of biomarker candidates for CVD and bone metabolism disorders.

## Integrated analysis

For further characterizing the interrelation between all 353 members of the interaction network represented by the largest sub-graph as given in Fig. 1 we extracted the following information for each single gene: The gene expression profile as found in CKD biopsy material published by Rudnicki *et al.*[36], as well as gene ontology terms on molecular process and function as provided by the gene ontology consortium [37, 38]. Additionally, we computed the transcription factor binding site profiles for each of the genes following *in silico* predictions as provided by the oPOSSUM tool [39, 40]. This procedure provides a list of transcription factors for each gene which appears to be involved in its differential regulation. Genes sharing transcription factors might be under similar expression control.

After assembling this set of properties for each of the 353 genes we computed pairwise correlations including the parameters gene expression, functional category and transcription factor modules. The rationale of this approach is the assumption that genes showing similarities on the level of these features might exhibit an increased likelihood for functional dependency in the context of cellular processes.

For characterizing the co-expression of two genes we used the Pearson correlation coefficient. Two genes exhibiting a high correlation coefficient of their expression profile are co-expressed on the level of differential gene expression. For expressing the pairwise similarity of two genes based on their gene ontology classification patterns the Dice coefficient for bit-strings was calculated. This string comparison measure determines the ratio of joint annotation within given categories and the total number of annotations in categories. High values of the Dice coefficient found for a given biomarker candidate pair indicate similarity on the level of functional categorization. The same measure was used for identifying the ratio of joint transcription factors indicating co-regulation between two genes. A meta-correlation based on the three single parameters was finally calculated for expressing functional dependency between elements of our biomarker candidate list.

**Fig. 2:**
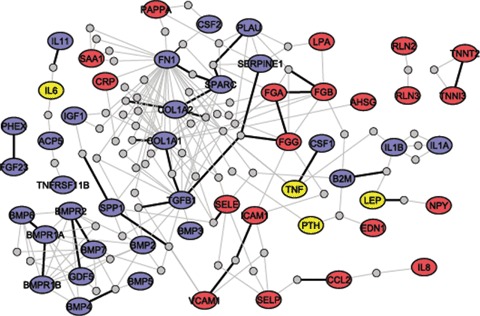
Detailed representation of the largest sub-graphs of the protein interaction network derived on the basis of 77 candidate markers. Only nearest neighbours having two edges to the CVD and bone marker candidates are shown. Protein nodes given in blue depict markers for bone metabolism disorders, protein nodes given in red hold markers of cardiovascular disease, and nodes given in yellow represent proteins reported in both diseases.

Applying this procedure provides correlation values for each interaction pair of the interaction graph given in Fig. 1. For subsequent analysis we focused on ‘strong’ pairwise interactions, defined as meta-correlation values which were found as at least one standard deviation above the mean value of all meta-correlation values for all pairs analysed. Figure 2 identifies these strong interactions as thick interaction lines.

Based on the protein interaction networks, and following the dependency measure expressed by the meta-correlation we identified two sub-networks, each connecting at least four of the reported biomarkers given in the initial list of 77 candidate biomarkers. The first sub-network holds the proteins fibronectin-1 (FN-1), collagen, type I, α2 (COL1A2), the plasminogen activator, urokinase (PLAU), and osteonectin (the secreted protein, acidic, cysteine-rich; SPARC). They were all reported to play a role in bone mineral disorders of patients with CKD. FN-1 is involved in various processes like cell adhesion and blood clotting, and has also been proposed as risk factor for arterial thrombosis [41]. SPARC regulates cell interactions with components of the extracellular matrix and is often found at sites of injury [42]. COL1A2 is mostly found in connective tissues and mutations in this gene regions were reported to lead to a variety of bone metabolism disorders including idiopathic osteoporosis, ostoegenesis imperfecta, or the Ehlers-Danlos syndrome [43, 44]. Besides its function in haemostasis PLAU is also involved in cell attachment and deformation of the extracellular matrix [45].

Members of the second sub-network are collagen, type I, α1 (COL1A1), the TGF-β_1_, the plasminogen activator inhibitor, also known as serpin peptidase inhibitor, clade E (SERPINE1), and the α, β and γ chains of fibrinogen (FG-α, FG-β, FG-γ). COL1A1 is like COL1A2 found in most connective tissues. TGF-β_1_ is a multifunctional protein involved in proliferation, differentiation, apop-totic processes, cell adhesion, and tissue remodelling [46]. SERPINE1 plasma concentrations are elevated in patients with increased risk of ischaemic cardiovascular events [47]. All three chains of fibrinogen are part of the network. After cleavage by thrombin, fibrin fibres form blood clots after vascular injury.

## Conclusion and outlook

We provide an interactome analysis approach to characterize the interplay of reported biomarker candidates for CVDs and bone metabolism disorders in CKD patients. Forty-six potential biomarkers for bone metabolism disorders and 31 potential biomarkers for CVD were identified in the literature and characterized with respect to biological function, gene expression in CKD, and known protein–protein interactions.

A majority of marker candidates for CVDs could be assigned to the functional category ‘immunity and defence’, whereas most of the bone metabolism genes were involved in skeletal and mesoderm development according to the PANTHER classification scheme. A category significantly enriched in both diseases was ‘signal transduction’ with various secreted signalling molecules being proposed as potential biomarkers. On the level of protein–protein interactions proteins involved in bone metabolism disorders were highly interlinked. The resulting IA was significantly higher than one would expect from randomly drawn gene lists. Biomarker candidates of CVDs were also closer connected as randomly generated gene lists although the statistical significance was not reached. The combined list of marker candidates from both diseases on the other hand was highly significant with around 68% of biomarkers forming the largest sub-graph of the overall protein–protein interaction network. Functional links of biomarkers proposed for CVD and bone metabolism disorders appear evident at least on this given level of data interpretation.

Of special note are the four potential biomarkers reported in both diseases, namely IL-6, PTH, LEP and TNF, as well as the three components of fibrinogen (FG-α, FG-β, FG-γ) building a major link between the two diseases as indicated by strong interactions based on the meta-correlation as depicted in Fig. 2. Although causal inference cannot be drawn form our data, the coincidence of features in both disease entities may potentially suggest choreographed action *via* a common pathway.

Integration of data from various sources for characterizing diseases has the potential to unravel novel pathophysiological mechanisms. As more and more tools become available for predicting protein–protein interactions based on protein domain information, the *in silico* validation of given protein candidates, but also identification of novel proteins playing a role in a given disease will become feasible [48, 49]. This development allows the analysis of the functional interplay among biomarker candidates, clearly providing routes towards identifying improved candidate markers.
